# Evaluation of Different Gene Prediction Tools in *Coccidioides immitis*

**DOI:** 10.3390/jof9111094

**Published:** 2023-11-09

**Authors:** Theo N. Kirkland, Sinem Beyhan, Jason E. Stajich

**Affiliations:** 1 Department of Medicine, Division of Infectious Disease, School of Medicine, University of California, La Jolla, CA 92093, USA; sbeyhan@health.ucsd.edu; 2 Department of Pathology, School of Medicine, University of California, La Jolla, CA 92093, USA; 3 Department of Infectious Diseases, J. Craig Venter Institute, La Jolla, CA 92037, USA; 4 Department of Microbiology and Plant Pathology, Institute for Integrative Genome Biology, University of California—Riverside, Riverside, CA 92521, USA; jason.stajich@ucr.edu

**Keywords:** *Coccidioides* spp., human pathogenic fungi, genomics, gene prediction

## Abstract

Gene prediction is required to obtain optimal biologically meaningful information from genomic sequences, but automated gene prediction software is imperfect. In this study, we compare the original annotation of the *Coccidioides immitis* RS genome (the reference strain of *C. immitis*) to annotations using the Funannotate and Augustus genome prediction pipelines. A total of 25% of the originally predicted genes (denoted CIMG) were not found in either the Funannotate or Augustus predictions. A comparison of Funannotate and Augustus predictions also found overlapping but not identical sets of genes. The predicted genes found only in the original annotation (referred to as CIMG-unique) were less likely to have a meaningful functional annotation and a lower number of orthologs and homologs in other fungi than all CIMG genes predicted by the original annotation. The CIMG-unique genes were also more likely to be lineage-specific and poorly expressed. In addition, the CIMG-unique genes were found in clusters and tended to be more frequently associated with transposable elements than all CIMG-predicted genes. The CIMG-unique genes were more likely to have experimentally determined transcription start sites that were further away from the originally predicted transcription start sites, and experimentally determined initial transcription was less likely to result in stable CIMG-unique transcripts. A sample of CIMG-unique genes that were relatively well expressed and differentially expressed in mycelia and spherules was inspected in a genome browser, and the structure of only about half of them was found to be supported by RNA-seq data. These data suggest that some of the CIMG-unique genes are not authentic gene predictions. Genes that were predicted only by the Funannotate pipeline were also less likely to have a meaningful functional annotation, be shorter, and express less well than all the genes predicted by Funannotate. *C. immitis* genes predicted by more than one annotation are more likely to have predicted functions, many orthologs and homologs, and be well expressed. Lineage-specific genes are relatively uncommon in this group. These data emphasize the importance and limitations of gene prediction software and suggest that improvements to the annotation of the *C. immitis* genome should be considered.

## 1. Introduction

The analysis of a genomic DNA sequence is a multistep process involving a number of bioinformatic algorithms. Once a high-quality DNA sequence is obtained, it must be assembled into contigs. In fungi and other eukaryotes, the next steps are to mask the repeat-rich regions, predict the intron and exon boundaries (ab initio gene prediction), and compare the predicted genes to expressed sequence tags (ESTs) and/or RNA-seq data from that organism, as well as comparing them to genes in other species. A large number of software packages and pipelines are available to perform this task, and different methods have been shown to result in differences in gene predictions [1,2,3,4,5,6]. This is particularly true in the prediction of lineage-specific genes, where, by definition, homology to genes in other species is very poor [2].

Funannotate (v1.8) is a software pipeline developed for gene prediction in fungi and other eukaryotes (https://github.com/nextgenusfs/funannotate, accessed on 6 November 2023). The pipeline consists of ab initio gene prediction methods including Augustus [7], SNAP [8], GlimmerHMM [9], CodingQuarry [10], and GeneMark-ES [11] for predicting intron/exon boundaries and a variety of tools for alignment of the predicted transcripts to RNA-seq data and Swissprot proteins. This approach has been used to predict genes in the *C. posadasii* Silveira strain, which has been sequenced to the chromosome level [12], as well as the WA_211 strain of *C. immitis* that was obtained from the soil in Washington State [13], and a number of isolates from other fungal species, including *Aspergillus fumigatus* [14].

*Coccidioides* spp. (*C. immitis* and *C. posadasii*) are dimorphic fungi that grow as mold in the desert soil in the Western hemisphere and form spherules in people and animals [15]. It is a serious human pathogen, frequently causing symptomatic infections and, less commonly, disseminated infections in immunocompetent people as well as the immunocompromised [16,17]. Pulmonary disease can be prolonged and debilitating, and disseminated infection, although uncommon, is frequently severe. One of the common types of disseminated infection is meningitis, which is fatal if not treated effectively and often leads to permanent neurologic sequalae [18]. For these and other reasons, this fungus is an important health problem in the endemic area.

The initial gene prediction analysis for the RS strain of *C. immitis* was performed in 2009 by the Broad Institute using their pipeline, which involved using manual review with more than 60,000 ESTs to predict gene expression [19,20]. RNA-seq data were not available at that time. This annotation has been used as a reference in the evaluation of most of the subsequently sequenced *C. immitis* isolates. Only four other *C. immitis* isolates have assembled genomes, and three of these were not sequenced to the same depth as *C. immitis* RS [20].

To evaluate the transcript prediction and functional annotation process of the *C. immitis* RS genome sequence, we have compared the gene predictions obtained from the Funannotate and Augustus pipelines to the current predictions, and the characteristics of the groups of genes have been investigated. This comparison is useful for evaluating the accuracy of the current gene predictions and serves as a case study for evaluating gene predictions obtained from a single genomic sequence analyzed by different methods.

## 2. Materials and Methods

### 2.1. Genomic Data

DNA sequence, gene predictions, and protein sequence for *C. immitis* RS (release-62) were obtained from FungiDB (https://fungidb.org/fungidb, accessed on 6 November 2023). The original annotation was designated the CIMG annotation. The protein sequence and nucleic acid sequence of the protein coding regions of 20 fungal genomes (Table S1), including at least one representative of the most common primary pathogenic human pathogens (except for *Coccidioides* spp.) were also obtained from FungiDB (release-62). Characteristics of the *C. immitis* RS CIMG annotation, including gene expression data, were also obtained from FungiDB. Gene expression data were calculated by the FungiDB staff from SRX10496548, SRX10496549, SRX10496550, SRX10496551, SRX10496552, and SRX10496553 (https://www.ncbi.nlm.nih.gov/sra/?term=C.%20immitis, accessed on 6 November 2023). They aligned the reads to the predicted genes using hisat2 [21] and counted the reads using hisat2-count to obtain transcripts per million (TPM) counts. This procedure was performed twice, using the -rf and -fr stranded modes to obtain strand-specific TPM.

### 2.2. Previously Published Data

Young spherule/mycelial fold change (FC) values have been previously published [22], as has the location of transposable elements in *C. immitis* RS [23]. Capped small RNA-seq data were published previously [24], and a table containing a summary of the data is included in the supplemental data (Table S2). Table S2 was made using scripts from homer.ucsd.edu (http://homer.ucsd.edu/homer/ngs/csRNAseq/index.html, accessed on 6 November 2023).

### 2.3. CIMG2 and Augustus Annotations

The Funannotate (v1.8) pipeline was run as previously described (https://funannotate.readthedocs.io/en/latest/predict.html#predict, accessed on 6 November 2023) [12,14]. RNA-seq data from NCBI were used as transcript evidence (SRX10496548, SRX10496549, SRX10496550, SRX10496551, SRX10496552, and SRX10496553) (https://www.ncbi.nlm.nih.gov/sra/?term=C.%20immitis, accessed on 6 November 2023). The results of this annotation were designated as the CIMG2 annotation. The output from the pipeline includes some functional annotations as well as gene predictions. The results are available at Zenodo [25].

Augustus gene predictions were performed using the online tool (https://bioinf.uni-greifswald.de/webaugustus/, accessed on 6 November 2023). The Augustus tool has previously been trained on *C. immitis*. RNA-seq data from NCBI were also provided (SRX10496548, SRX10496549, SRX10496550, SRX10496551, SRX10496552, and SRX10496553) (https://www.ncbi.nlm.nih.gov/sra/?term=C.%20immitis, accessed on 6 November 2023). The results of this annotation were designated the Augustus annotation. The results are available at Zenodo [25].

### 2.4. Software

Gffcompare v0.12.2 was obtained from http://ccb.jhu.edu/software/stringtie/gffcompare.shtml (accessed on 6 November 2023) [26]. The CIMG annotation was the reference sequence, and the CIMG2 and Augustus predictions were the queries. Matches between the CIMG annotation and either the CIMG2 or the Augustus predictions were scored as common gene predictions. OrthoVenn2 was used online (https://orthovenn2.bioinfotoolkits.net/home, accessed on 6 November 2023) to compare the CIMG-predicted protein sequences to the CIMG2-predicted protein [27]. Orthologs were determined with an e-value less than 10^−10^ and an inflation value of 1.5. Mmseqs2 Version 14-7e284 was obtained from https://github.com/soedinglab/MMseqs2 (accessed on 6 November 2023) [28]. The easy-search function was used to search for homologs in a database of predicted fungal proteins from 20 species (Table S1). Predicted genes with more than five matches with an e-value of less than 10^−50^ were scored as positive for protein homology. Nucleotide homologs of the coding regions of a selection of 1000 CIMG genes with more than 200 orthologs were compared to 1000 selected CIMG-unique orthologs with mmseqs2. The target database was the coding nucleotide sequences of the same 20 fungal species described in Table S1. Predicted genes with more than five matches with an e-value of less than 10^−50^ were scored as positive for nucleotide homology. Bedtools v2.30.0 was obtained from https://bedtools.readthedocs.io/en/latest/ (accessed on 6 November 2023 [29]. Proximity to transposable elements (TE) was determined using the bedtools window function, with the window set to 250 base pairs upstream or downstream of the TE.

Kallisto v0.48.0 was obtained from https://pachterlab.github.io/kallisto/ (accessed on 6 November 2023) [30]. Gene expression of gene predictions was obtained by mapping RNA-seq data (SRX10496548, SRX10496549, SRX10496550, SRX10496551, SRX10496552, and SRX10496553) to the Funannotate (CIMG2) gene predictions with Kallisto. Sense and antisense TPM values were obtained using the -rf stranded option.

Data analysis was performed in R version 4.2.3. The Chi-Square test was used to determine the statistical significance of differences of proportions. The Wilcoxon test was used to determine the statistical significance of differences in medians. The Kolmogotov–Smirnov test was used to determine the statistical significance of differences in distributions. 

## 3. Results

We compared annotations using three methods and explored the resulting predictions of two methods in detail. The original annotation (denoted CIMG), the Funannotate annotation (denoted CIMG2), and the Augustus annotation (denoted Augustus) were compared using the gffcompare tool, which performs a stringent comparison of gene structures [26]. Three-quarters of the currently predicted CIMG genes were found in at least two of the three predictions. Slightly over half of the predictions had an identical intron/exon structure (Table S3). A total of 2563 genes were found in all three predictions; 2902 conserved genes were found only in the CIMG and the CIMG2 prediction; and 2017 genes were found only in the CIMG and the Augustus prediction.

**Figure 1:**
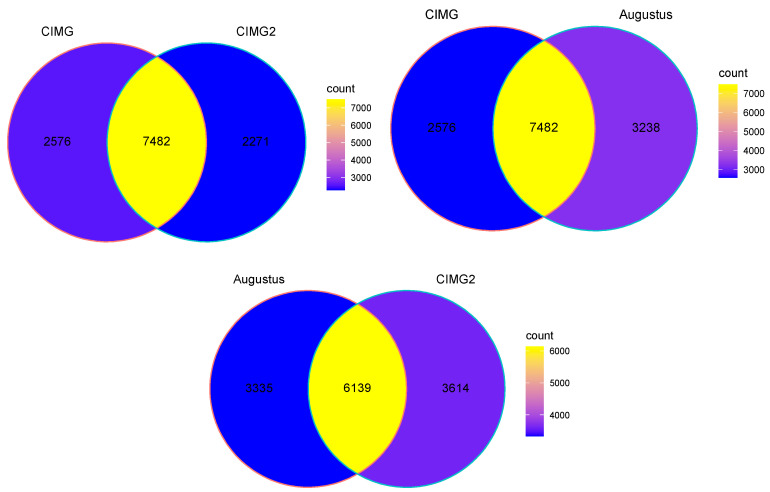
Overlapping and unique gene predictions using three different methods. Venn diagrams depicting overlapping and unique genes (as determined by gffcompare) in the three annotations.

Examining the entire sets of transcripts (Figure 1), it is clear that all three methods predict overlapping sets of genes, but a significant number of non-overlapping genes are also predicted. Although the Augustus gene prediction tool is part of the Funannotate pipeline, other gene prediction tools are also included in the Funannotate pipeline, which probably accounts for the differences in gene predictions between these two methods.

**Figure 2:**
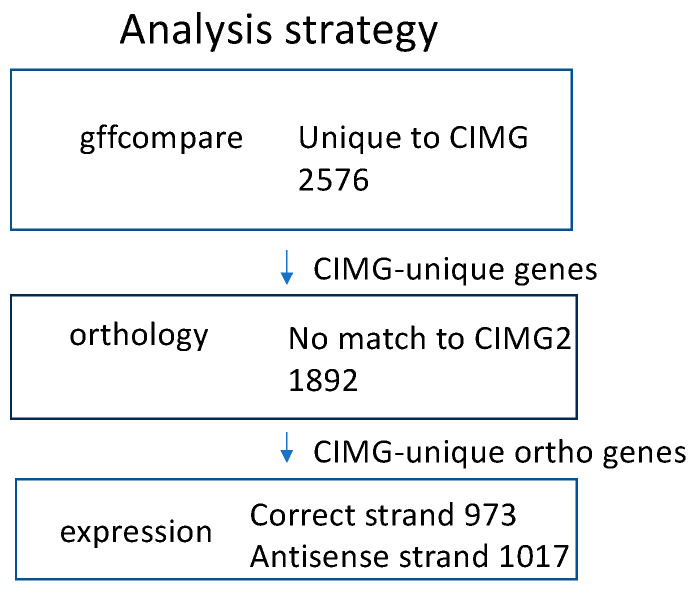
Strategy for analyzing CIMG genes. Expression was defined as more than 5 TPM.

We have focused on the CIMG and CIMG2 annotations because the Augustus algorithm provides no functional annotation for the gene predictions. Protein homology is another approach to comparing gene annotations. The predicted proteins that were found only in the CIMG or CIMG2 annotations by gffcompare were tested for orthology to the alternative prediction using OrthoVenn2. The CIMG-unique genes that lacked CIMG2 orthologs were designated CIMG-unique ortho genes, and the CIMG2-unique predictions that lacked CIMG orthologs were designated CIMG2-unique ortho genes. The overall strategy for identifying CIMG-unique transcripts is shown in Figure 2. An identical strategy was used to identify CIMG2-unique genes.

### 3.1. Comparing All CIMG-Predicted Genes to CIMG-Unique Genes

The characteristics of predicted CIMG-unique transcripts compared to all CIMG-predicted transcripts and the CIMG-unique ortho-predicted transcripts are shown in Table 1. 

The CIMG-unique and CIMG-unique ortho-predicted transcripts are shorter, much more likely to lack meaningful functional annotations, and have many fewer orthologs compared to the whole set of CIMG-predicted transcripts. A total of 671 (36%) of the CIMG-unique ortho-predicted transcripts have no orthologs, which indicates that they are only found in the RS strain of *C. immitis*, and 1589 (84%) have less than 10 orthologs, which is consistent with being lineage-specific. A total of 73% of the total number of lineage-specific genes are in the CIMG-unique set. In addition, 90% of the CIMG-unique ortho set of predicted transcripts are lineage-specific, which represents 67% of the total number of lineage-specific genes in the total CIMG annotation. In contrast, only 640 (8.56%) of the CIMG genes that are found in at least two annotations are lineage-specific.

Homology searches were also performed using MMseqs2. Genes with more than five protein alignments with a value less than 10^−50^ were classified as having homologs. A relatively small proportion of the CIMG-unique and a very small proportion of the CIMG-unique ortho sets of genes met this criteria, which is also consistent with the conclusion that many are lineage-specific. Consistent with this idea, the manual inspection of 200 randomly selected CIMG-unique genes found that only 41 (21%) had synteny with genes in other fungi. Another approach to assessing homology uses nucleotide comparisons. Only 0.6% of the CIMG-unique ortho-predicted genes had more than 5 homologs using nucleotide sequence, compared to 57% of the CIMG-predicted genes with more than 200 orthologs, emphasizing the poor conservation of the unique genes.

**Figure 3:**
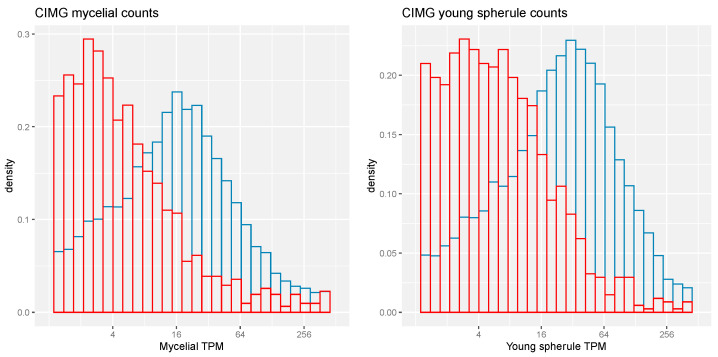
RNA-seq expression of CIMG and CIMG-unique ortho genes. **Left**—CIMG sense-stranded mycelial counts. **Right**—CIMG sense-stranded young spherule counts. Red—CIMG-unique ortho genes. Blue—all CIMG genes. Kolmogotov–Smirnov and Wilcoxon tests, *p* values < 0.05.

Differential expression of CIMG genes in mycelia compared to spherules has been published [22]. All three sets of transcripts contain similar fractions of differentially expressed transcripts, which suggests that some of each group may play a functionally important role in differentiation in spherules. Stranded RNA-seq expression data are also available [24]. The number of CIMG-unique ortho genes with significant sense-strand RNA-seq counts was substantially lower than was seen in the total set of CIMG transcripts in both the mycelial and spherule phases of the organism (Figure 3).

**Figure 4:**
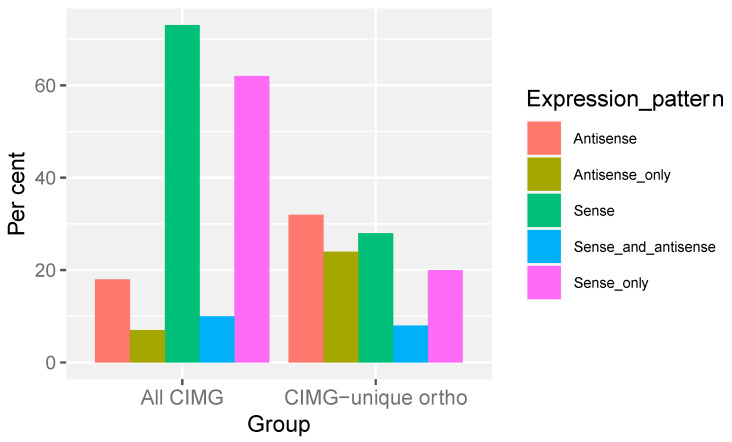
Expression pattern. Genes with more than 10 TPM were scored as positive. Sense and antisense TPM were obtained as described in Methods.

Transcription from both the sense and antisense strands occurs in many fungi [31,32,33], including *C. immitis* [24]. Antisense transcripts play a role in gene regulation via several mechanisms. The percentages of genes with significant sense and antisense expression in the predicted CIMG-unique ortho and total CIMG gene sets are shown in Figure 4.

The majority of the total set of CIMG-predicted genes was expressed on the sense strand, and most were expressed exclusively on the sense strand, in contrast to the CIMG-unique ortho set, where a much smaller proportion were expressed on the sense strand (expression was defined as more than 10 TPM). More CIMG-unique ortho-predicted genes were only expressed in the antisense orientation than in the set of all CIMG genes. This finding raises the question of whether CIMG-unique ortho genes with significant antisense expression are valid gene predictions.

**Figure 5:**
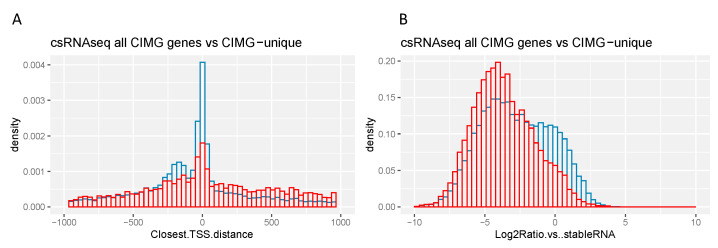
csRNA-seq. (**A**) Distance between observed TSS and predicted start site. (**B**) Ratio of stable RNA to csRNA. Red—CIMG-unique ortho genes. blue—all CIMG genes. Smirnov and Wilcoxon tests, *p* values < 0.05.

Transcription start sites (TSS) have also been mapped to the CIMG-predicted genes with the capped small RNA-seq technique (csRNA-seq) [24]. This approach reveals that there is usually more than one TSS associated with a predicted gene, and over 95% of the time, TSS were found both upstream and downstream of a predicted gene. When all predicted CIMG genes were compared to CIMG-unique ortho genes, these data showed that the number of TSS per predicted gene was different—3.34 for the unique ortho genes and 2.54 for the total set of genes. In addition, the distribution of the distance of TSS from the CIMG annotation-predicted gene start site was different (Figure 5).

The csRNA-seq sequences in the CIMG-unique ortho-predicted genes are less likely to cluster very close to the predicted gene start sites, and the secondary cluster upstream that is seen in the whole set of predicted genes is not observed (Figure 5). Another metric that is different between the two sets of genes is the ratio of csRNA-seq transcription to stable RNA. CIMG-unique ortho-predicted genes tend to have a smaller ratio of stable RNA (determined by RNA-seq) to TSS transcription (determined by csRNA-seq).

**Figure 6:**
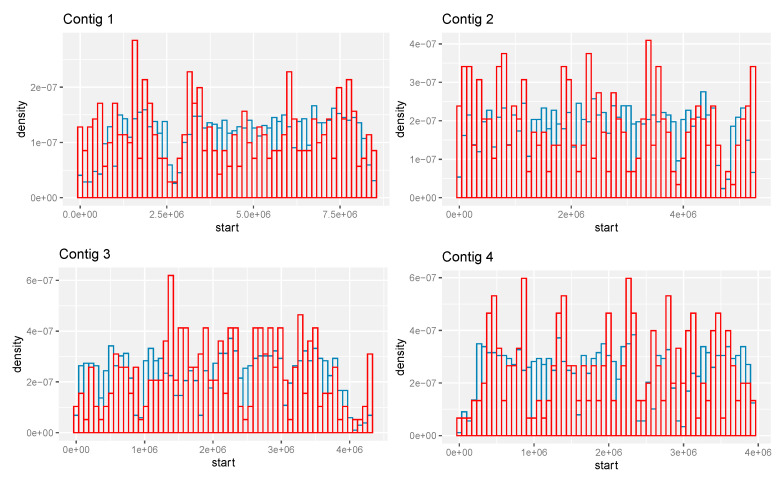
Location of predicted genes on contigs 1–4. Red—CIMG-unique ortho genes. Blue—all CIMG genes.

The spatial distribution of the predicted CIMG-unique ortho-predicted genes on the contigs is also somewhat different than the total set of predicted genes. The CIMG-unique ortho set tends to cluster, and prominent clusters are at the left ends of contigs 1 and 2, which are relatively gene-poor in the CIMG annotation (Figure 6).

In addition, the CIMG-unique (23%) and CIMG-unique ortho (27%) genes are more likely to be within 250 bp up- or downstream of a transposable element compared to all genes (11%). This association may play a role in gene prediction because masking repetitive sequences is an important part of the gene prediction pipeline.

CIMG-unique genes with at least 50 counts in mycelia, young spherules, or mature spherules that were also differentially expressed (fold change > 1.5 or <−1.5) were designated CIMG-unique, highly expressed, differentially expressed genes. There were 55 CIMG-unique highly expressed, differentially expressed genes. These were manually compared to the RNA-seq data in the FungiDB GBrowse. Only 26 of these genes had reasonably good matches between the RNA-seq data and the predicted intron/exon structure by visual inspection. 

### 3.2. Comparing All CIMG2-Predicted Genes to CIMG2-Unique Genes

**Figure 7:**
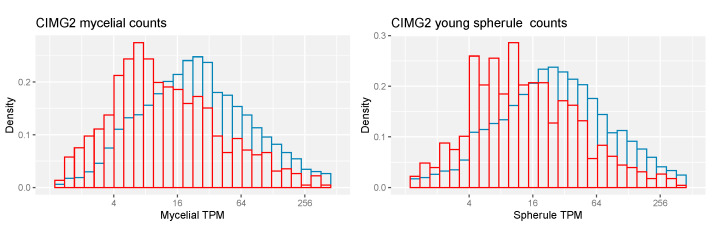
RNA-seq expression CIMG2 and CIMG2-unique ortho-predicted genes. **Left**—CIMG2 mycelial counts. **Right**—CIMG2 young spherule counts. Red—CIMG2-unique ortho genes. Blue—all CIMG2 genes. Kolmogotov–Smirnov and Wilcoxon tests, *p* values < 0.05.

There are 2271 CIMG2-unique genes and 1066 CIMG2-unique ortho genes (Table 2). A smaller percentage of CIMG2-unique and CIMG2-unique ortho genes has a meaningful annotation than the whole set of genes, and a lower percentage has homologs in other fungi. The unique proteins are also shorter than the whole set. The expression of CIMG2-unique ortho genes is lower than the total set of genes (Figure 7).

Less than 5% of both the total set of CIMG2 genes and the CIMG2-unique ortho-predicted genes were significantly expressed on the antisense strand.

## 4. Discussion

Automated gene prediction is a very challenging task. A recent study compared the performance of five different algorithms with a very large database of genome sequences from many eukaryotes and found that Augustus was the most accurate, but it only predicted the correct amino acid sequence 75% of the time. This study did not include Funannotate and had few fungi in the test dataset [5]. It is especially difficult to accurately predict genes that are lineage-specific because there are so few homologs to help predict gene structure [2].

The initial gene predictions of the RS strain of *C. immitis* were made based on ESTs, but they have not been re-evaluated since 2009, despite the development of newer computational approaches for gene prediction and some RNA-seq data to guide intron/exon predictions. Funannotate is a pipeline that incorporates several different ab initio methods for intron/exon predictions as well as methods for a comparison of the predicted CDS to experimental data, and it has been successfully used for gene predictions in a variety of fungi [12,13,14]. Augustus is also a relatively accurate pipeline with both ab initio and homology components [7]. It is based on a generalized Hidden Markov Model that calculates a probabilistic model that predicts intron and exon boundaries. This program was enhanced to include external data, such as EST, RNA-seq, or protein data, which increased the accuracy of the predictions [7]. It has been trained on *C. immitis* and is available as a web application. This study compares different methods of predicting genes for one genome assembly. Although the majority of predicted genes were identical or very similar, about a quarter of gene predictions are different, as measured by gffcompare, which is a stringent test of similarity [26]. Similar observations have been reported by previous studies, although many of them have compared gene prediction techniques using a large number of genomes. In the case of *C. immitis* predictions, CIMG-unique and CIMG-unique ortho-predicted genes were significantly different than the total set of CIMG-predicted genes. They were shorter, less likely to have a functional annotation, and more likely to be lineage-specific. Lineage specificity was defined as a small number of orthologs, but the unique genes also had a much smaller number of homologs than the entire set of CIMG-predicted genes. Almost all of the CIMG-unique ortho genes had poor nucleotide matches to other pathogenic fungi. More than 60% of the total number of CIMG-predicted lineage-specific genes were in the CIMG-unique ortho set. These observations may indicate that the CIMG-unique genes are more rapidly evolving than the entire set of CIMG-predicted genes. An alternative hypothesis is that the CIMG prediction contains a number of incorrectly predicted genes. These two hypotheses are not mutually exclusive. 

The CIMG-unique and CIMG-unique ortho gene sets were not as well expressed as all CIMG-predicted genes. In addition, the percentage of CIMG-unique genes with significant sense transcription was only half of that in the total CIMG gene predictions, while the percentage of genes with significant antisense expressions was twice as high. Antisense expression occurs in all types of organisms and plays a variety of roles, including regulation of transcription and translation [31,34,35]. Nonetheless, the difference in the antisense prediction of CIMG-unique gene predictions and all genes predicted by the CIMG method is striking. However, it is difficult to determine whether the “antisense” is actually antisense because that designation depends on valid gene predictions. An analysis of csRNA-seq data for CIMG-unique predicted genes showed that the TSS was less likely to coincide with the predicted gene start site compared to all CIMG-predicted genes. Furthermore, TSS transcription is less likely to be associated with stable RNA. These results also suggest that some of the CIMG-unique gene predictions may be erroneous.

The CIMG-unique genes are differently spatially distributed than all CIMG genes and are more likely to be near a transposable element. This may be important because masking repetitive DNA content is an important part of gene prediction, and errors in this task may result in errors in gene prediction. Differential expression of putative genes in mycelia versus spherules would suggest that those predicted genes are valid. About 20% of the CIMG-unique and CIMG-unique ortho genes are differentially expressed, which is not different than the value seen in the whole set of predicted genes. However, there were only 55 CIMG-unique, highly expressed, differentially expressed genes. The most reliable bioinformatic annotation process is visual inspection in a gene browser to map RNA-seq data to the predicted gene structure. The CIMG-unique, highly expressed, differentially expressed genes were compared to the RNA-seq in a genome browser. Some of the predicted genes had different splicing in mycelia and spherules, a finding that might be biologically important. Only 26 of these genes had reasonably good matches between the RNA-seq data and the predicted intron/exon structure. However, the significance of this finding is questionable since very few RNA-seq studies are available [22,24,36]. Furthermore, some genes may be expressed in unusual conditions, at low levels, or transiently, so deciding that a predicted transcript is not valid is difficult. 

The genes that are found in at least two of the three prediction methods have a number of characteristics. They are more likely to have a functional gene annotation, to have many orthologs, and to have many homologs in other fungi. In addition, they tend to be expressed at a higher level and longer. All this suggests that genes predicted by three methods are more likely to be highly conserved, and these gene predictions have a high probability of being valid. Lineage-specific genes are relatively uncommon in this group.

CIMG2-unique transcripts that are only predicted by the Funannotate pipeline also tend to be shorter, less likely to have a functional annotation or homologs, and less well expressed. Based on this analysis, we cannot conclude that the Funannotate pipeline is more accurate than the initial CIMG annotation. However, these results suggest that more accurate gene predictions are needed for *C. immitis*. Hopefully, obtaining a complete genome sequence and a thorough annotation, which have been achieved with *C. posadasii*, will be helpful [19]. In addition, obtaining the genome sequence of more strains and much more RNA-seq data should help improve gene annotation.

## Figures and Tables

**Table 1:** Characteristics of sets of genes predicted by the CIMG pipeline.

	Total	% ^a^	CIMG-Unique	% ^b^	CIMG-Unique Ortho	% ^c^
Total	10,058		2576		1892	
Hypothetical product description	3318	32.99	1905	73.95	1645	86.95
Any PFAM domain	6077	60.42	523	20.30	63	3.33
Molecular function GO annotation	4449	44.23	407	15.80	38	2.01
Linage-specific (< 10 orthologs)	2381	23.67	1741	67.59	1589	83.99
Number of orthologs ^d^	204		2		1	
Genes with homologs ^e^	6388	63.51	518	20.11	30	1.59
Differential expression ^f^	2009	19.97	581	22.55	406	21.46
Protein length ^g^	347		155		131	

^a^ Compared to the total number of genes; ^b^ compared to the number of CIMG-unique genes; ^c^ compared to the number of CIMG-unique ortho genes; ^d^ median number of orthologs; ^e^ genes with more than five protein blast matches as defined in methods; ^f^ Differential expression defined as fold change values of young spherules/mycelia of >1.5 or <−1.5, ^g^ median values.

**Table 2:** Characteristics of sets of genes predicted by the CIMG2 pipeline.

		% ^a^	CIMG2-Unique	% ^b^	CIMG2-Unique Ortho	% ^c^
Total	9753		2271		1066	
“Hypothetical protein” product description	7541	77.32	1988	87.54	959	89.96
Any PFAM domain	6425	65.88	918	40.42	301	28.24
Genes with homologs ^d^	8712	89.33	740	32.58	325	30.49
Protein length ^e^	389		168		137	

^(a)^ Compared to the total number of genes; ^(b)^ compared to the number of CIMG2-unique genes; ^(c)^ compared to the number of CIMG2-unique ortho genes; ^(d)^ genes with more than five protein blast matches as defined in methods; ^(e)^ median.

## Data Availability

Data are available at Zenodo: [25].

## Supplementary Materials

The following supporting information can be downloaded at: https://www.mdpi.com/article/10.3390/jof9111094/s1. Table S1: Fungal_species_S1.docx; Table S2: gffcompare_results_S2.docx; Table S3: csRNA-seq_results_S3.xlsx.
