# Supplementary material for: Evaluation of Different Gene Prediction Tools in Coccidioides immitis
Source: J Fungi (Basel). 2023 Nov 9;9(11):1094. doi: 10.3390/jof9111094 (PMC10672684; doi:10.3390/jof9111094)
Supplement: Supplementary file 1 [file jof-09-01094-s001.zip › jof-2670523-supplementary-Table S1. Fungal_species_S1.pdf]

Aspergillus fumigatus A1163
Aspergillus fumigatus Af293
Blastomyces dermatitidis ATCC_26199
Blastomyces dermatitidis ER-3
Blastomyces gilchristii SLH14081
Candida albicans WO1
Candida auris strain 6684
*Cryptococcus neoformans var. neofor*mans B-3501A
Cryptococcus neoformans var. grubii H99
Histoplasma capsulatum G184AR
Histoplasma capsulatum H88
Neurospora crassa OR74A
Paracoccidioides brasiliensis Pb03
Paracoccidioides lutzii Pb01
Pneumocystis jirovecii RU7
Saccharomyces cerevisiae S288C
Sporothrix schenckii 1099-18
Talaromyces marneffei ATCC_18224
Trichoderma reesii QM6a
Uncinocarpus reesii 1704
