# Supplementary material for: Evaluation of Different Gene Prediction Tools in Coccidioides immitis
Source: J Fungi (Basel). 2023 Nov 9;9(11):1094. doi: 10.3390/jof9111094 (PMC10672684; doi:10.3390/jof9111094)
Supplement: Supplementary file 1 [file jof-09-01094-s001.zip › jof-2670523-supplementary-Table S2. gffcompare_results_Table_S2.pdf]

Table S2

Results of gffcompare analysis

Match	Number	%
=	5948	79.50
c	1145	15.30
k	217	2.90
m	269	3.60
Total	7482	74.38

Legend: Number of matched predictions comparing CIMG, CIMG2 and Augustus predictions. The total number CIMG predicted genes is 10058. = indicates exact intron, exon match; c indicates included in reference (intron compatible); k indicates containment of reverse (reverse containment); m indicates contained introns, all introns matched or retained.
